# Ischemic heart disease among subjects with and without chronic obstructive pulmonary disease – ECG-findings in a population-based cohort study

**DOI:** 10.1186/s12890-015-0149-1

**Published:** 2015-12-04

**Authors:** Ulf Nilsson, Bengt Johansson, Berne Eriksson, Anders Blomberg, Bo Lundbäck, Anne Lindberg

**Affiliations:** Department of Public Health and Clinical Medicine, Division of Medicine, Umeå University, Umeå, Sweden; Krefting Research Centre, Institute of Medicine, Sahlgrenska Academy, University of Gothenburg, Gothenburg, Sweden; Department of Public Health and Clinical Medicine, Division of Medicine, University Hospital of Northern Sweden, Umeå, 90185 Sweden

**Keywords:** Comorbidity, Epidemiology, Coronary disease, Respiratory diseases

## Abstract

**Background:**

Cardiovascular comorbidity in COPD is common and contributes to increased mortality. A few population-based studies indicate that ischemic electrocardiogram (ECG)-changes are more prevalent in COPD, while others do not. The aim of the present study was to estimate the presence of ischemic heart disease (IHD) in a population-based COPD-cohort in comparison with subjects without COPD.

**Methods:**

All subjects with obstructive lung function (COPD, *n* = 993) were identified together with age- and sex-matched controls (non-COPD, *n* = 993) from population-based cohorts examined in 2002–04. In 2005, data from structured interview, spirometry and ECG were collected from 1625 subjects. COPD was classified into GOLD 1–4 after post-bronchodilator spirometry. Ischemic ECG-changes, based on Minnesota-coding, were classified according to the Whitehall criteria into probable and possible IHD.

**Results:**

Self-reported IHD was equally common in COPD and non-COPD, and so were probable and possible ischemic ECG-changes according to Whitehall. After excluding subjects with restrictive spirometric pattern from the non-COPD-group, similar comparison with regard to presence of IHD performed between those with COPD and those with normal lung-function did neither show any differences. There was a significant association between self-reported IHD (*p* = 0.007) as well as probable ischemic ECG-changes (*p* = 0.042), and increasing GOLD stage. In COPD there was a significant association between level of FEV_1_ percent of predicted and self-reported as well as probable ischemic ECG-changes, and this association persisted for self-reported IHD also after adjustment for sex and age.

**Conclusion:**

In this population-based study, self-reported IHD and probable ischemic ECG-changes were associated with COPD disease severity assessed by spirometry.

**Electronic supplementary material:**

The online version of this article (doi:10.1186/s12890-015-0149-1) contains supplementary material, which is available to authorized users.

## Background

The prevalence of chronic obstructive pulmonary disease (COPD) is approximately 10 %, and there is still a significant under-diagnosis with up to 80 % of the cases not yet identified by healthcare [1–3]. COPD is associated with several comorbidities whereof cardiovascular disease (CVD) is the most common [4] and both of these conditions are among the leading causes of death worldwide [5, 6]. The associations between COPD and CVD are complex; besides sharing common risk factors such as smoking and aging [7, 8], also systemic inflammation is suggested as a possible link between the conditions [9, 10].

Among cardiovascular diseases, ischemic heart disease (IHD) is of particular interest. . According to the WHO, globally IHD was responsible for 7.5 million out of the total 17.4 million CVD deaths in 2012. Hospital-based studies have shown that IHD is common among subjects with COPD [11, 12] and there is an increased risk for death after a myocardial infarction among those with COPD compared to those without [13–15]. A recently published review clearly shows that IHD worsens the disease progress as well as prognosis among subjects with COPD [5, 6, 16]. However, the under-diagnosis of COPD most probably contributes to an underestimation of the real impact of IHD among subjects with COPD in the general population. Simple diagnostics signs of IHD in COPD, such as ischemic findings on electrocardiogram (ECG), have hardly been evaluated in population-based studies.

The aim of this population-based study was to estimate the prevalence of self-reported ischemic heart disease and ischemic ECG changes among subjects with COPD in comparison with subjects without obstructive lung function impairment, divided into normal lung function and restrictive pattern. Our hypothesis was that the prevalence of IHD would be higher among subjects with COPD compared with subjects with normal lung function and that the prevalence of IHD would increase with COPD disease severity, as assessed by spirometry.

## Methods

### Study population

Four population-based cohorts from the Obstructive Lung Disease in Northern Sweden (OLIN) studies were re-examined during 2002–2004. All subjects with obstructive lung function impairment (COPD) were identified (*n* = 993) together with an age- and sex-matched reference population without obstructive lung function impairment. The study population (*n* = 1986) has since 2005 been invited to annual examinations with a basic program including spirometry and structured interview [17].

This study is based on data collected in 2005 when ECG recordings were performed in addition to the basic program. In total, 1641 participated whereof 1625 had complete data on spirometry, structured interview and ECG. The structured interview questionnaire included questions validated in national and international studies [18–21]. The Regional Ethics Committee at Umeå University approved the study (approval number 04-045 M), which was carried out according to the declaration of Helsinki. Written informed consent for participation in the study was obtained from all participants.

### Definitions

Self-reported IHD was based on data from the structured interview, and was defined as any history of angina pectoris, myocardial infarction, coronary artery bypass surgery (CABG) or percutaneous coronary intervention. Hypertension was defined by affirmative answer to the question “Do you have high blood pressure?” Body mass index (BMI) was calculated and classified in four groups (underweight <20, normal 20–24.9, overweight ≥ 25–29.9 and obesity ≥ 30 kg/m^2^). Smoking habits were classified as non-smoker, ex-smoker (stopped since at least 12 months) and smokers, as well as by pack years.

### Electrocardiogram

Standard twelve-lead ECG’s were recorded before spirometry, on subjects in the supine position and after sufficient rest. Two independent physicians analyzed all ECG recordings according to the Minnesota code (MC) and both of them were blinded to COPD status and spirometry values. ECG-based IHD was defined in accordance to the Whitehall criteria as probable IHD; major Q/QS wave (MC 1.1–1.2) and left bundle branch block (LBBB) (MC 7.1.1). Possible IHD was defined as minor Q/QS wave (MC 1.3), ST segment depression (MC 4.1–4.3), and T wave items (MC 5.1–5.3) [22].

### Lung function tests

Lung function test was performed in accordance with the American Thoracic Society guidelines [23], following ECG registration, using a dry volume spirometer, the Vicatest 5 (Gebr. Mijnhardt B.V., Odijk, Netherlands). Vital capacity (VC) was defined as the highest value of forced vital capacity (FVC) and slow vital capacity (SVC) pre- or post- reversibility test. Reversibility test was performed with Ventoline® 4x0.2 mg if FEV_1_/VC <0.70 or if FEV_1_ < 80 % of predicted value. COPD was defined as FEV_1_/VC <0.70 using the best values of VC and FEV_1_ pre- or post-reversibility test. Severity of airflow limitation in COPD was classified according to the Global Initiative for Chronic Obstructive Lung Disease (GOLD) document; GOLD 1–4 based on FEV_1_ % predicted [24]. The OLIN reference values, based on healthy non-smokers, were applied [25, 26].

The reference population without obstructive lung function impairment, defined as FEV_1_/VC ≥0.70, was further divided into subjects with normal lung function (NLF), FEV_1_/VC ≥0.70 and VC >80 %, and subjects with restrictive pattern on dynamic spirometry defined as FEV_1_/VC ≥0.70 and VC <80 % of predicted.

### Statistics

Statistical calculations were performed using the Statistical Package for the Social Sciences (SPSS) software version 21.0 (IBM, Armonk, NY, USA). Missing data on smoking habits and self-reported cardiovascular disease in a total of 4 subjects were collected from data from the previous and/or following year’s examinations. The chi-square test was used for categorical variables and bivariate comparisons. Mantel-Haenszel test for trend and Fischer’s exact test was used where appropriate. Independent sample *t*-test and ANOVA were used to compare means. Association between the continuous variable FEV_1_ percent of predicted and self-reported IHD respectively probable and possible ischemic ECG changes according to Whitehall was analyzed by bivariate logistic regression with each of the IHD variables as dependent variable. Analyses were also performed in similar models adjusting for age and sex. P-values <0.05 were considered statistically significant.

A cross-sectional analysis of COPD and non-obstructive lung function based on spirometry in 2005 was performed. A further analysis was performed in which the subjects had to fulfill the spirometric criteria of COPD vs. non-COPD not only in 2005 but also at baseline, i.e. at recruitment in 2002–04 and defined as “stable COPD” and “stable non-COPD”. Those with non-COPD were further divided into restrictive pattern and NLF.

## Results

### Characteristics of the study population in 2005

Among all subjects with complete data on spirometry, interview and ECG in 2005, 634 subjects out of 1625 fulfilled the spirometric criteria of COPD. Basic characteristics are presented in Additional file 1. The burden of tobacco smoking, assessed as smoking habits as well as pack years, was significantly higher among COPD-subjects.

There was no significant difference in the prevalence of self-reported IHD when comparing non-COPD and COPD, although myocardial infarction was significantly more common among COPD-subjects and CABG was more common among non-COPD-subjects. Ischemic ECG changes, analyzed separately or categorized according to Whitehall, were similarly common in non-COPD and COPD (Additional file 2).

### Characteristics of stable non-COPD and stable COPD

#### Comparing subjects with normal lung function and subjects with restrictive pattern

Among subjects classified as non-COPD, FEV_1_/VC ≥0.70, both at baseline and in 2005 (*n* = 757), 145 subjects had a restrictive pattern on dynamic spirometry. Subjects with restrictive pattern had higher BMI and higher prevalence of diabetes, hypertension, angina, myocardial infarction and arrhythmias compared to subjects with NLF (Table 1).

**Table 1:** Basic characteristics. Comparing subjects with normal lung function (NLF) vs. restrictive pattern and normal lung function vs. COPD. Analyses were performed among subjects “stable non-COPD” (NLF and restrictive pattern) and “stable COPD); at baseline and in 2005 (significant values in bold)

Categories	Variables	NLF *n* = 612	Restrictive pattern *n* = 145	P^c^	COPD *n* = 576	P^d^
Sex						
	Women, n (%)	292 (47.7)	60 (41.4)	0.17	243 (42.2)	**0.06**
Age						
	Mean (SD)	64.8 (11.0)	68.6 (10.1)	**<0.001**	66.6 (10.6)	**0.004**
BMI						
	Mean (SD)	27.1 (4.0)	28.4 (4.4)	**0.001**	26.3 (4.1)	**0.001**
	Underweight <20, n (%)	6 (1.0)	4 (2.8)		22 (3.8)	
	Normal 20–24.9, n (%)	194 (31.7)	27 (18.8)		204 (35.4)	
	Overweight 25–29.9, n (%)	290 (47.4)	68 (47.2)		259 (45.0)	
	Obese ≥30, n (%)	122 (19.9)	45 (31.3)		91 (15.8)	
Smoking habits						
	Pack years, mean (SD)	6.5 (10.3)	6.8 (11.3)	0.74	17.0 (16.2)	**<0.001**
	Current smoker, n (%)	71 (11.6)	13 (9.0)		207 (35.9)	
	Ex smoker, n (%)	234 (38.2)	52 (35.9)		241 (41.8)	
	Non smoker, n (%)	307 (50.2)	80 (55.2)		128 (22.2)	
Comorbidities^a^						
	Diabetes, n (%)	47 (7.7)	26 (17.9)	**<0.001**	49 (8.5)	0.60
	Hypertension, n (%)	197 (32.2)	69 (47.6)	**0.001**	198 (34.4)	0.42
	Angina pectoris, n (%)	67 (10.9)	27 (18.6)	**0.01**	70 (12.2)	0.521
	Myocardial infarction, n (%)	14 (2.3)	12 (8.3)	**0.001**	32 (5.6)	**0.004**
	CABG, n (%)	18 (2.9)	7 (4.8)	0.25	10 (1.7)	0.17
	PCI, n (%)	8 (1.3)	5 (3.4)	0.07	8 (1.4)	0.90
	Arrhythmias, n (%)	44 (7.2)	20 (13.8)	**0.01**	46 (8.0)	0.60
	Reported IHD^b^, n (%)	77 (12.6)	32 (22.1)	**0.005**	93 (16.1)	0.08

^a^Based on interview data. ^b^Self-reported IHD, includes any of angina pectoris, myocardial infarction, CABG and PCI

P^c^ comparison between normal lung function and restrictive pattern P^d^ comparison between normal lung function and COPD

#### Comparing subjects with normal lung function and subjects with COPD

Basic characteristics comparing subjects with FEV_1_/VC ratio <0.70 both at baseline and in 2005 (*n* = 576) and subjects with NLF at both occasions (*n* = 612) are given in Table 1. Subjects with COPD were older, had a lower mean BMI, and the burden of tobacco smoking was higher considering both current smoking and pack years (Table 1). The distribution of COPD by GOLD classification was: GOLD 1, *n* = 221 (38.4 %), GOLD 2, *n* = 308 (53.5 %) and GOLD 3–4, *n* = 47 (8.2 %).

The prevalence of self-reported IHD was 16.1 % among COPD and 12.6 % among subjects with NLF (*p* = 0.08). Myocardial infarction was significantly more common among those with COPD compared with NLF (Table 1).

### Ischemic ECG findings

#### Comparing subjects with normal lung function and subjects with COPD

The prevalence of ischemic ECG changes, defined as Q/QS wave, ST segment depression, T wave items and left bundle branch block, was similar among subjects with COPD and NLF, also when ischemic ECG changes were divided into major, intermediate and minor (Table 2).

**Table 2:** Ischemic ECG changes comparing subjects with normal lung function and COPD

			NLF *n* = 612	COPD *n* = 576	P
Ischemic ECG changes					
	Q-Waves	Any, n (%)	49 (8.7)	48 (8.3)	0.64
		Major Q/QS, n (%)	20 (3.4)	24 (4.2)	0.42
		Minor Q/QS, n (%)	29 (4.9)	24 (4.2)	0.66
	ST-segment depressions	Any, n (%)	39 (6.4)	33 (5.7)	0.23
		Major, n (%)	4 (0.7)	4 (0.7)	0.94
		Intermediate, n (%)	20 (3.3)	18 (3.1)	0.88
		Minor, n (%)	15 (2.5)	11 (1.9)	0.52
	T- wave items	Any, n (%)	98 (16.0)	100 (17.4)	0.53
		Major, n (%)	2 (0.4)	1 (0.2)	1.00
		Intermediate, n (%)	44 (7.9)	45 (8.7)	0.64
		Minor, n (%)	52 (9.2)	54 (9.4)	0.56
	Bundle branch block	LBBB^a^, n (%)	10 (1.9)	11 (2.3)	0.68
Whitehall criteria					
	Ischemic heart disease	Any, n (%)	140 (22.9)	142 (24.7)	0.47
		Probable^b^, n (%)	27 (5.4)	35 (6.1)	0.19
		Possible^c^, n (%)	113 (19.3)	107 (18.6)	0.85

^a^Left bundle branch block. ^b^Including Major Q/QS and LBBB. ^c^Including Minor Q/QS, any ST-segment depression and any T-wave item

When ischemic ECG changes were analysed in groups according to the Whitehall criteria, “any IHD” had similar prevalence among subjects with NLF and COPD, 22.9 % and 24.7 % respectively. The subgroups “probable IHD” and “possible IHD” were also equally prevalent among subjects with COPD and subjects with NLF (Table 2).

#### Ischemic heart disease in relation to COPD severity

**Fig. 1:**
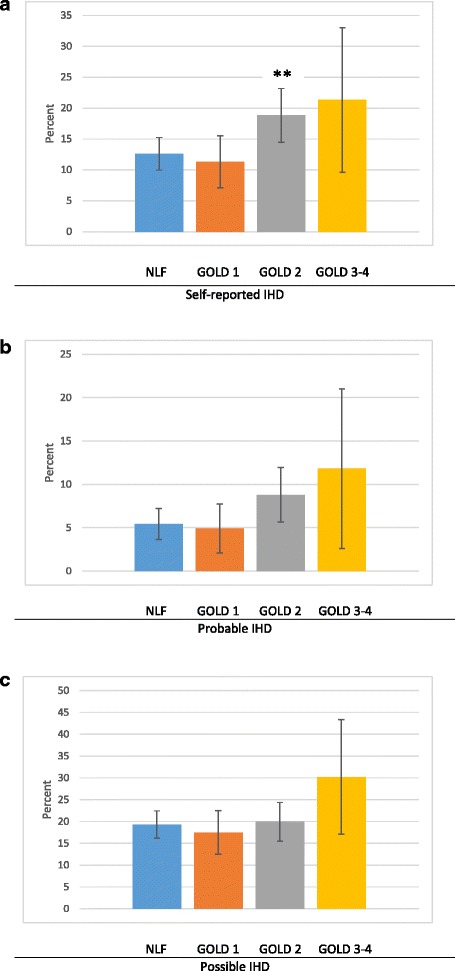
Ischemic heart disease categorized as (**a**) Self-reported IHD and (**b**) Ischemic ECG changes according to Minnesota coding categorized as probable Whitehall criteria and (**c**) Possible Whitehall criteria among subjects with normal lung function (NLF), COPD GOLD 1, 2 and 3–4, with 95 % confidence intervals. Significance is shown with **p ≤ 0.01 (NLF compared with GOLD 1, 2 and 3–4, respectively). Observe that **a**, **b** and **c** have different scales on the y-axis

The prevalence of self-reported IHD was higher in GOLD 2 compared with in subjects with NLF (Fig. 1a), and the test for trend NLF -- GOLD 3–4 was significant (*p* = 0.007). When comparing the prevalence of ischemic ECG-changes in each of the GOLD stages with the prevalence among those with NLF, there were no differences within any of the Whitehall groups (Fig. 1b, c). Test for trend in NLF -- GOLD 3–4 was significant in subjects with Whitehall probable IHD (*p* = 0.042), but not among Whitehall possible IHD (*p* = 0.355).

In a logistic regression analysis, FEV_1_ percent of predicted value was significantly associated with self-reported IHD and probable ischemic ECG-changes according to Whitehall, but not possible ischemic ECG changes according to Whitehall (Table 1). When adjusting for age and sex, the association between FEV_1_ percent of predicted value and self-reported IHD persisted, while the association with ischemic ECG-changes corresponding to Whitehall probable IHD was lost (Table 3).

**Table 3:** Logistic regression analysis of self-reported ischemic disease, probable and possible ischemic heart disease by Whitehall criteria in relation to FEV_1_ percent of predicted value among subjects with COPD. (significant values in bold)

	Reported IHD		Probable IHD		Possible IHD	
	OR	95 % CI	OR	95 % CI	OR	95 % CI
FEV1 % pred	**0.13**	**0.03–0.48**	**0.10**	**0.01–0.79**	0.41	0.11–1.48
FEV1 % pred^a^	**0.22**	**0.06–0.85**	0.24	0.03–1.97	0.55	0.15–2.04

^a^Adjusted for age and sex

## Discussion

In this cross sectional population based study, self-reported as well as ECG changes of ischemic heart disease (IHD) according to Whitehall were equally prevalent among subjects with COPD and those with normal lung function (NLF). There was a significant association between increasing presence of self-reported IHD as well as probable ECG changes of IHD, according to the Whitehall criteria, in relation to COPD disease severity, both classified according to GOLD stage as well as assessed as FEV_1_ percent of predicted. The significant relationship between FEV_1_ percent of predicted and self-reported IHD but not probable ECG-findings remained after adjustment for age and sex.

According to a recent review, the prevalence of coronary heart disease (CHD) in different COPD populations varies between 4.7 % and up to 60 % [27]. Since the recruitment of study population, as well as the definitions of CHD and COPD, vary between the studies, it is difficult to compare the results. In a Danish population-based study [28], the prevalence of IHD was 8.6 % in non-COPD and increased in COPD by GOLD stage 1, 2 and 3–4; 12.1 %, 14.9 % and 13.7 % respectively; the corresponding prevalences of Q-wave (major + minor) were 2.7 % in non-COPD, and 3.3 %, 6.2 % and 6.9 % by GOLD stages. Overall, self-reported heart disease and presence of corresponding ECG-findings were markedly more common in our study compared with in the Danish survey. One possible explanation for the observed difference is that although the COPD classification in the Danish study was based on spirometry, they used pre-bronchodilator values, i.e. not following current guidelines recommending post-bronchodilator spirometry for the diagnosis of COPD. Other studies have shown that the use of pre-bronchodilator spirometry misclassify up to 27 % of non-obstructive subjects as obstructive, which might contribute to the lower IHD prevalence [29]. Further, the Danish study was a close to 25-year follow-up of the large-scale Copenhagen City Heart study (with the addition of a smaller random sample of younger adults) and a healthy survivor effect can thus be expected to have affected the results. In a population-based study using the Burden of Obstructive Lung Disease (BOLD) survey, close to 100 cases of COPD were identified based on post-bronchodilator spirometry and they found no increased risk for self-reported cardiovascular disease or hypertension in either of the GOLD-stages [30]. In general, population based data on CHD among subjects with COPD identified by post-bronchodilator spirometry is mainly self-reported, and other clinical findings of CHD, such as ECG-changes, have rarely been evaluated.

A population-based COPD-cohort, such as in our study, will include a majority of subjects with GOLD 1 and 2 and only a low number of subjects with GOLD 3–4 [1]. Self-reported IHD as well as ischemic ECG-findings according to the Whitehall criteria clearly increased by GOLD stage and were considerably more common in GOLD 3–4 than among subjects with mild COPD. Even though our study include a large COPD-cohort, almost comparable to that of the National Health and Nutrition Examination Survey (NHANES) I [31], the distribution of GOLD stages contributes to a lack of power to show statistical significance due to the small proportion of severe/very severe COPD in a population based study, as also demonstrated by the quite large confidence intervals in Fig. 1. However, each of the GOLD stages include rather wide ranges of FEV_1_ % predicted, and the continuous variable FEV_1_ % predicted may be useful when it comes to evaluating events in relation to disease severity. The found positive associations between level of FEV_1_ among COPD-subjects and self-reported IHD as well as probable ischemic ECG-changes according to the Whitehall criteria, thus further support a relationship between IHD and COPD disease severity. Interpretation of the results is that higher FEV_1_ is protective, consequently, the presence of IHD increase the lower FEV_1_ is, i.e. by increasing COPD disease severity. Furthermore, the observed high prevalence of ischemic ECG-changes in more severe COPD disease is comparable with findings from studies using hospitals records including severe stages of COPD [11, 12, 32].

It has also been observed that subjects with COPD have increased arterial stiffness [33], which is considered a marker of early atherosclerosis and a risk factor for development of CVD. In a recent review it was even suggested that measurements of arterial stiffness should be included in routine health care to assess the risk for CVD among subjects with COPD [34]. We have previously shown that central arterial stiffness is higher in GOLD 3–4 than in non-COPD [35] also in a population based study, which is in line with the current findings of IHD being most prevalent in GOLD 3–4. The clinical implication may be that not only non-invasive measurements of arterial stiffness, but also ECG may be of value when evaluating risk for and presence of cardiovascular disease when diagnosing subjects with COPD. The underlying pathological mechanism by concomitant COPD and IHD is, however, not fully understood, but may be related to systemic inflammation and endothelial dysfunction [5, 6, 16], and in addition high on-treatment platelet reactivity has been discussed as reason for the observed worsened prognosis among subject with concomitant COPD and IHD [5, 6, 36].

The original design of the study defined cases and sex and age-matched controls based on spirometry (COPD and non-COPD) in 2002–04. It has been described in other COPD studies that a proportion of the study population will transfer mainly from COPD to non-COPD but also *vice versa* during follow up [37]. To address this potential bias, data from 2005 is presented as a straight cross-sectional analysis defining COPD and non-COPD by current spirometry, but also by presenting data when the subjects had to fulfill the spirometric criteria of either COPD or non-COPD not only in 2005 but also at baseline, i.e. at recruitment in 2002–04. Among subjects with non-obstructive pulmonary function, also subjects with a restrictive pattern on dynamic spirometry were included. Restrictive pattern has in previous studies been shown to associate with metabolic disorders such as diabetes, as well as a higher prevalence of IHD [38] or risk for cardiovascular disease [30]. Thus we divided non-COPD into NLF and restrictive spirometric pattern and compared subjects with COPD with those having NLF.

A limitation of this study is that the self-reported burden of comorbid conditions, in this study specifically ischemic heart disease, may be affected by recall bias as well as misclassification since interview data were not verified by medical records, even though literature has shown a good agreement between self-reported diabetes, hypertension and myocardial infarction, but not heart failure [39]. Further, a 12-lead ECG only gives a short glimpse of the electrical activity of the heart, and has limited sensitivity and specificity for detecting ischemic myocardial events. Different ischemic ECG changes, for example ST segment changes and those involving the T-waves, have great variations in sensitivity and specificity, are dynamic and may thus vary over time [40, 41]. However, the strength of this epidemiological study is the large sample size, the distribution of disease severity representative for COPD in the general population [31, 42], the use of standardized post-broncho-dilator spirometry for defining COPD in accordance with the GOLD document [24], and double blinded Minnesota coded ECGs. To the best of our knowledge, this is the first population-based study addressing ischemic heart disease among subjects with COPD using validated and generally recommended classifications of ECG-findings and COPD.

## Conclusions

In this population-based study, ischemic heart disease was equally common among subjects with COPD and those with normal lung function. Among subjects with COPD, there was a significant association between higher prevalence of self-reported ischemic heart disease and the results also indicate that there is an association between ECG changes of ischemic heart disease and increasing disease severity, as assessed by level of FEV_1_. A longitudinal follow-up is important to evaluate the prognostic value as well as progression of the observed ECG-findings among subjects with and without COPD.

## Additional files

Additional file 1:
**Basic characteristics, reported respiratory symptoms and comorbidities of all subjects (** ***n*** **= 1625), comparing non-COPD and COPD.** (PDF 163 kb) Additional file 2:
**Ischemic ECG changes in all subjects (** ***n*** **= 1625), comparing non-COPD and COPD.** (PDF 92 kb)

## Abbreviations

BMI: body mass index
CABG: coronary artery bypass graft
COPD: chronic obstructive pulmonary disease
CVD: cardiovascular disease
ECG: electrocardiogram
FEV_1_: forced expiratory volume during one second
FVC: forced vital capacity
GOLD: global initiative for chronic obstructive lung disease
IHD: ischemic heart disease
LBBB: left bundle branch block
MC: Minnesota code
NLF: normal lung function
PCI: percutaneous coronary intervention
SVC: slow vital capacity
VC: vital capacity
