# Supplementary material for: Ischemic heart disease among subjects with and without chronic obstructive pulmonary disease – ECG-findings in a population-based cohort study
Source: BMC Pulm Med. 2015 Dec 4;15:156. doi: 10.1186/s12890-015-0149-1 (PMC4670536; doi:10.1186/s12890-015-0149-1)
Supplement: Additional file 1: — Basic characteristics, reported respiratory symptoms and comorbidities of all subjects ( n = 1625), comparing non-COPD and COPD. (PDF 163 kb) [file 12890_2015_149_MOESM1_ESM.pdf]

Additional file 1. Basic characteristics, reported respiratory symptoms and comorbidities of all subjects (n=1625), comparing non-COPD and COPD (significant values in bold).

Categories	Variables	Non-COPD n=991	COPD n= 634	P
Sex				
	Women, n (%)	470 (47.4)	270 (42.6)	0.06
Age				
	Years, mean (SD)	65.4 (11.2)	67.1 (10.6)	**<0.01**
Body constitution				
	Body mass index, mean (SD)	27.4 (4.3)	26.4 (4.1)	**<0.001**
	Underweight <20, n (%)	16 (1.6)	24 (3.8)	
	Normal 20-24.9, n (%)	294 (29.7)	220 (34.7)	
	Overweight 25–29.9, n (%)	450 (45.5)	285 (45.0)	
	Obese ≥30, n (%)	230 (23.2)	105 (16.6)	
Smoking habits				
	Pack years, mean (SD)	7.2 (11.2)	16.1 (16.1)	**<0.001**
	Current smoker, n (%)	123 (12.4)	212 (33.5)	
	Ex smoker, n (%)	399 (40.3)	261 (41.2)	
	Never smoker, n (%)	469 (47.3)	160 (25.3)	
Comorbidities[1]				
	Diabetes, n (%)	108 (10.9)	58 (9.1)	0.26
	Arterial hypertension, n (%)	352 (35.5)	224 (35.3)	0.94
	Angina pectoris, n (%)	122 (12.3)	80 (12.6)	0.86
	Myocardial infarction, n (%)	34 (3.4)	36 (5.7)	**0.03**
	CABG, n (%)	39 (3.9)	12 (1.9)	**0.02**
	PCI, n (%)	18 (1.8)	11 (1.7)	0.90
	Arrhythmias, n (%)	86 (8.7)	55 (8.7)	0.99
	Reported IHD[2], n (%)	146 (14.7)	108 (17.0)	0.21

[1]Based on interview data. [2]Reported IHD includes any of angina pectoris, myocardial infarction, CABG and PCI.
