# Supplementary material for: Ischemic heart disease among subjects with and without chronic obstructive pulmonary disease – ECG-findings in a population-based cohort study
Source: BMC Pulm Med. 2015 Dec 4;15:156. doi: 10.1186/s12890-015-0149-1 (PMC4670536; doi:10.1186/s12890-015-0149-1)
Supplement: Additional file 2: — Ischemic ECG changes in all subjects ( n = 1625), comparing non-COPD and COPD. (PDF 92 kb) [file 12890_2015_149_MOESM2_ESM.pdf]

Additional file 2. Ischemic ECG changes in all subjects (n=1625), comparing non-COPD and COPD

			Non-COPD n=991	COPD n= 634	P
Ischemic ECG changes					
	Q-Waves	Any, n (%)	78 (7.9)	54 (8.5)	0.72
		Major Q/QS, n (%)	33 (3.5)	26 (4.3)	0.42
		Minor Q/QS, n (%)	45 (4.7)	28 (4.6)	0.93
	ST-segment depressions	Any, n (%)	69 (7.0)	36 (5.7)	0.30
		Major, n (%)	6 (0.6)	4 (0.7)	0.97
		Intermediate, n (%)	34 (3.6)	21 (3.4)	0.86
		Minor, n (%)	21 (3.1)	11 (1.8)	0.13
	T- wave items	Any, n (%)	173 (17.5)	110 (17.4)	0.96
		Major, n (%)	3 (0.4)	1 (0.2)	1.00
		Intermediate, n (%)	74 (8.3)	49 (8.6)	0.85
		Minor, n (%)	96 (10.5)	60 (10.3)	0.90
	Bundle branch block	LBBB[1], n (%)	18 (2.1)	15 (2.8)	0.41
Whitehall criteria					
	Ischemic heart disease	Any, n (%)	240 (24.2)	158 (24.9)	0.75
		Probable[2], n (%)	48 (6.0)	40 (7.8)	0.22
		Possible[3], n (%)	192 (20.4)	118 (19.9)	0.81

[1]Left bundle branch block. [2]Including Major Q/QS and LBBB. [3]Including Minor Q/QS, any ST-segment depression and any T-wave item
